# Writing Patient Engagement Effectively Into Grant Applications: Practical Tips for Grant Writers

**DOI:** 10.1111/hex.70547

**Published:** 2026-01-14

**Authors:** Lisa D. Hawke, Katie Upham, Hajar Seiyad, Mary Rose van Kesteren

**Affiliations:** ^1^ Centre for Addiction and Mental Health Toronto Ontario Canada; ^2^ University of Toronto Toronto Ontario Canada

1

Engaging people with lived/living experience and caregivers in research (also known as ‘patient engagement’ or ‘patient and public involvement’) has many benefits to research, to the community, and to the people involved in the process [1]. Engagement is increasingly valued by many funding bodies [2, 3] as being best practice when possible. However, funding barriers have been described as getting in the way of authentic engagement [4]. It is therefore important that grant writers optimise their descriptions of the engagement components of their protocols, making engagement a strength of their applications. Researchers have many grant writing recommendations, courses, and professional development materials available to them to support them in their grant‐writing endeavours [5, 6]. Yet, there is minimal concrete, specific guidance on how to embed lived/living experience and caregiver engagement into grant applications effectively [7]. With this editorial, we aim to address this gap.

The engagement description in a grant application serves to clarify the applicants' engagement methods for their own purposes and sets them out to be judged by a team of peer reviewers, who are the gatekeepers to funding. Although rare, some funders include people with lived/living experience and caregivers as peer reviewers [8], who are well positioned to evaluate proposed engagement strategies. Whether lived/living experience peer reviewers are included or not, the way engagement is described in the protocol can influence the funding decision. If the engagement is poorly described and peer reviewers fear tokenistic engagement approaches, the application may be less likely to be funded. Regardless of page limits and tight application requirements, engagement can be well described in any protocol, and should be described together with lived/living experience and caregiver co‐applicants or collaborators when possible.

Through our experience in writing and reviewing grant applications, we have come to recognise when engagement is well described, making it a strength of the application, versus when it is poorly described, raising fears of tokenistic, performative engagement. Based on this experience, we propose a number of recommendations to guide grant writers in effectively describing engagement in their grant applications (see Box 1). These recommendations can be generally followed regardless of the format of the funding call, to the extent possible within the limits of the application guidelines. Below, we describe an overview of how engagement might be described poorly in a grant application, which we contrast with the description of a robustly described engagement plan. In doing so, our goal is to equip researchers to optimise the engagement components of their grant applications to maximise the chances of funding, thereby increasing the amount of patient‐engaged research being conducted.

Box 1: **Grant writing tips for describing lived/living experience and caregiver engagement.**

1 **Dos**

- Embed engagement throughout the protocol.
- Acknowledge the importance of lived/living and caregiver expertise in the project.
- Include a mention of engagement in important sections like the objective, the impact, and/or the relevance statement.
- Mention and cite at least one model or framework of authentic engagement practices.
- Know and cite your funder's engagement guidelines, if available.
- Describe the engagement plan (how many people, how often, in what format, etc.).
- Describe a range of tasks that people with lived/living experience and caregivers will complete.
- Describe accessibility measures and budget for them, if there is room to do so.
- Budget enough hours to allow for meaningful engagement and emergent interests and ensure the budget matches the described engagement plan.
- If possible, engage at the grant‐writing stage, leverage lived/living experience and caregiver feedback to shape the project, and describe this in the application.
- Include people with lived/living experience and caregivers on the applicant team or a letter of support from them, ensuring that the individuals chosen align with the population of interest for the study.
- Demonstrate that the project lead and/or other team members have the knowledge, training, and commitment to conduct engagement in a meaningful manner.
- Ensure that the engagement plan is consistent throughout all application components.

**Don'ts**

- Begin thinking about and doing engagement after the grant application is funded.
- Limit the mention of engagement to the last page of the protocol.
- Mention engagement without any supporting guidelines, models, or frameworks.
- Overlook the funder's guidelines around engagement.
- Mention and plan for the engagement of only one person, one voice.
- Engage the people with lived/living experience and caregivers who are easiest to reach, but who do not reflect the population of interest.
- Describe an engagement plan that is more substantial than the budget requested to cover the work.
- Focus only on the academic expertise of team members, overlooking the importance of the lived/living expertise of other team members.
- Use ‘buzz words’ around engagement, without truly understanding them or believing in them.
- Overpromise and underdeliver.

## 2 Poorly Described Engagement Plans

When peer reviewing grant applications, all too often, we have seen that the plan to engage people with lived/living experience and caregivers in the research is described on the last page of the application, often in a superficial statement that engagement will occur. The grant writers do not appear to be aware of the funding institution's perspective on engagement, if any. Citations to academic documents describing engagement practices, models, and frameworks are generally absent, missing out on the opportunity to express that best practices in engagement will be followed. Engagement descriptions lack depth and specificity regarding the type of engagement that will occur. The concrete tasks that people with lived/living experience and caregivers will complete are undisclosed. For clues about the number and length of engagement sessions, one must review the budget line indicating honoraria to pay those lived/living experience members, which is often limited. There may be inconsistencies in the description of engagement, often between the protocol and the corresponding budget, but also within the protocol itself. People with lived/living experience and caregivers are not included as co‐applicants or collaborators on the project and there is no letter of support expressing their intention to contribute. If they are included, their lived experience might not align with the population of focus of the grant application, which is key to increasing the relevance of the results. No engagement appears to have occurred prior to the submission of the grant application. Importantly, the applicants do not appear to have training or expertise in engagement. They seem to be just using a few engagement ‘buzz words,’ without truly understanding what they mean.

In this context, a peer reviewer might question the authenticity and meaningfulness of the engagement process, and any lived/living experience and caregiver peer reviewer might react with frustration and retraumatization. The engagement process might be perceived as merely a tokenistic check‐box aiming to meet funder requirements, rather than reflecting a legitimate interest and commitment on the part of the applicant team. Regardless of the original intentions of the grant writers, in this case, engagement is unlikely to be evaluated as a strength of the application that would support a positive funding decision.

## 3 Well‐Described Engagement Plans

In contrast, some grant applications express engagement plans that are compelling and exciting to read. They highlight engagement as a core value of the research in a way that is consistent with the funding institution's mandate, instructions, and values and that convinces peer reviewers of the authenticity of the engagement plan. Engagement appears front and centre and a peer reviewer can be confident that the researchers have the commitment and skills to engage people with lived/living experience and caregivers in a fulsome way, embodying inclusive, respectful, sincere, and meaningful engagement.

A grant application with a well‐written engagement plan has a number of characteristics. Notably, engagement is present throughout the application and is embedded as a core value. Applicant teams refer to and cite models, frameworks, or best practices in engagement, including any offered by the funder, and they make the commitment to follow them. A concrete engagement structure is provided, including the number, frequency, and length of meetings; the number of people with lived/living experience and caregivers who will be engaged; and specific tasks that they will complete throughout the course of the project, from the beginning to the end. The plan is feasible, believable, and consistent throughout the project. ‘Co‐’ language is used throughout the grant, with the grant writers indicating that a study tool or product will be co‐produced, co‐selected, or co‐created. Engagement may even be embedded directly in the objective statement, with objectives such as ‘In direct collaboration with people with lived/living experience and caregivers, we aim to…’. This carries forward to the impact and relevance statements and the conclusions of the application. If the grant includes a research implementation phase, the same lived/living experience and caregiver partners are engaged to help translate the findings to real‐world practice. The associated budget demonstrates the commitment to paying people with lived/living experience and caregivers a living wage or higher for all meetings, presentations, and asynchronous work time, plus travel expenses to encourage equity, with some room for unplanned emergent contributions. Accessibility and diversity considerations are described. More than one person with lived/living experience might be included as knowledge users or collaborators, or have provided a letter of support, and they reflect the population of focus of the grant application. Their expertise is highlighted and valued. Ideally, some initial engagement will have already occurred to refine the research question, and the lived/living and caregiver experience feedback that helped shape the grant application is described. The applicants have demonstrated that they have expertise in engagement, genuinely care about ensuring equitable collaboration, and truly understand the engagement process, or have brought on a reliable team member with this complementary expertise.

A grant application that follows some or all of these recommendations will seem more sincere to peer reviewers, demonstrating a fulsome and meaningful engagement plan. Engagement becomes a key strength of the application and one factor driving peer reviewers toward a decision to recommend funding.

## 4 Conclusions

We are committed to supporting research that embeds lived/living experience throughout the research process, from idea generation to knowledge mobilisation. We believe that embedding meaningful engagement across the research sector will create more relevant research, more meaningful experiences for those engaged and for the researchers, a more rigorous evidence base, and ultimately a stronger healthcare system that is more reflective of the needs of those they serve, while building capacity for all research team members. However, in order for engaged research to become dominant across health research, it needs to be encouraged by funding institutions—and to be successfully funded. We hope these recommendations will help grant writers emphasise engagement as a strength of their applications. We further hope that this will increase the funding success rate for well‐engaged research teams, helping to raise the bar in research to make strong engagement plans the gold standard, and ultimately leading to health systems benefits.

## Author Contributions

**Lisa D. Hawke:** conceptualisation, writing – original draft, funding acquisition. **Katie Upham, Hajar Seiyad, Mary Rose van Kesteren:** conceptualisation, writing – review and editing.

## Ethics Statement

The authors have nothing to report.

## Consent

This editorial was co‐authored by people with lived/living experience and grant peer review experience.

## Conflicts of Interest

The authors declare no conflicts of interest.

## Data Availability

The authors have nothing to report.
